# On entropy research analysis: cross-disciplinary knowledge transfer

**DOI:** 10.1007/s11192-018-2860-1

**Published:** 2018-08-06

**Authors:** R. Basurto-Flores, L. Guzmán-Vargas, S. Velasco, A. Medina, A. Calvo Hernandez

**Affiliations:** 1 Instituto Politécnico Nacional, Unidad Profesional Interdisciplinaria en Ingeniería y Tecnologías Avanzadas, 07340 Ciudad de México, Mexico; 2 Departamento de Física Aplicada, Universidad de Salamanca, 37008 Salamanca, Spain; 3 Instituto Universitario de Física Fundamental y Matemáticas (IUFFyM), Universidad de Salamanca, 37008 Salamanca, Spain

**Keywords:** Complex networks, Scientific data, Computational science, Applied physics

## Abstract

Our aim is to illustrate how the thermodynamics-based concept of entropy has spread across different areas of knowledge by analyzing the distribution of papers, citations and the use of words related to entropy in the predefined Scopus categories. To achieve this, we analyze the Scopus papers database related to entropy research during the last 20 years, collecting 750 K research papers which directly contain or mention the word entropy. First, some well-recognized works which introduced novel entropy-related definitions are monitored. Then we compare the hierarchical structure which emerges for the different cases of association, which can be in terms of citations among papers, classification of papers in categories or key words in abstracts and titles. Our study allowed us to evaluate, to some extent, the utility and versatility of concepts such as entropy to permeate in different areas of science. Furthermore, the use of specific terms (key words) in titles and abstracts provided a useful way to account for the interaction between areas in the category research space.

## Introduction

During the last decades, the increasing trend in the number of scientific publications has attracted the attention of investigators with the purpose of mapping the general structure and interrelationships among different areas of scientific knowledge (Bollen et al. 2009; Guevara et al. 2016). Bibliometric techniques have become a powerful tool allowing qualitative and quantitative studies of scientific and technological publications mainly based on citation and content analysis. The extent of such techniques is also reflected by thermodynamic concepts like entropy itself (Prathap 2011b, a). These approaches have also been widely used to account for the impact of research fields, of scholars, particular publications and/or authors, journal rankings, quality indexes for authors, institutions, publications, and so on Garfield (1972), Subelj et al. (2014), Chatterjee et al. (2016), Moreira et al. (2015), Radicchi and Castellano (2012), Waltman (2016). In a complementary way, bibliometric methods can be used to study the history of scientific topic evolution, from its appearance until its extinction or merging with other topics (Mryglod et al. 2016). As a consequence, interlinkages between different fields can be analyzed. Particularly, citation and coauthorship classification analyses have revealed that knowledge transfer can be evaluated in an indirect way (Rinia et al. 2002).

Along the above line, the main goal of this paper is to investigate the cross-disciplinary usage of the entropy concept, one of the most important and subtle concepts, not only in Thermodynamics, but also in Physics and other fields of Science. Entropy has attracted attention in many subcategories of the Sciences, not only in Thermodynamics, but also in Physics and other fields of the natural sciences, and beyond. Consequently, Entropy can be found in many subcategories as used in bibliographic databases of the (natural) Sciences, as well as in the Arts, Humanities, and Social Sciences, since the emergence of early concepts by Carnot (linked to performance/efficiency of heat devices) (Carnot 1824) and Clausius (linked to reversibility/irreversibility) (Clausius 1862), until its present-day formulation based on Shannon’s entropy (Shannon 1948), mostly linked to information of complex systems of very different natures and scales

The common use of concepts (boundary objects) by different groups or communities has been studied from the perspective of sociology, information science and knowledge management. We warn that in the case of entropy, its use in different areas of knowledge, especially in science and engineering, is characterized by a rigidity that contrasts with the necessary flexibility that characterizes a boundary object (Star and Griesemer 1989). Interestingly, entropy and related concepts have been used to suggest a connection between the evolution of thermodynamic and bibliometric systems (Prathap 2011b).

Moreover, the entropy concept and its presence in many different research categories across all fields of Sciences seems to be a powerful tool to investigate how many different fields of knowledge are linked as a straightforward consequence of cross-disciplinary interaction. The cross-disciplinary works are a common matter not just in the case of entropy, but in science in general. From such a study it is possible to point out the relationship among different areas of science as stated by different studies (Boyack et al. 2005; Leydesdorff and Rafols 2009; Klavans and Boyack 2009). The present work does not try to represent the whole Map of Science, but rather to study a specific sample. This paper (with 750K research files from the Scopus database) is mainly focused on the time evolution of key related concepts, entropies with specific given names; and more important, on the structural interaction of the research space coming from citations among different categories according to the Scopus classification, and co-occurrence of key words in abstracts and titles.

Next, we present the main characteristics of the dataset. After that, we study the evolution in time and the papers per category of five main entropies with a given name which are associated, respectively, to five seminal papers. This is complemented with the evolution of some key entropy-related words. Finally, our results focus on interactions in the research space accounting for citations and paper classification by using natural language analysis. Some technical details about the methodology are summarized in “Appendix”.

## General description of the dataset

Scopus is one of the largest indexing databases providing information about academic publications from many different areas of knowledge, including social and economic sciences. This database is recognized for providing easy access to article information and links to citations and references for documents, among many others features. The Scopus website permits a search mode based on a specific query, which can be author, name, keyword, etc. We collected papers from Scopus with the *entropy* search term. Regarding the particular information for publications or articles, each paper has 22 different fields of associated metadata including title, abstract, authors, affiliation authors, cited by count, document type, volume, issue, ISSN, ISBN, and publication date (Elsevier 2017a). Data are limited from January first of 1996 to December 31, 2015. The database was harvested during October 2016. The total number of tracked papers rises to 750 K. We filtered the data to exclude papers inside the Trade Publication category as this is used as a leftover container with many documents that may lack the precision of indexed journals.

In accordance with information provided by *Scopus* (Elsevier 2017b), a general classification of journals is given in terms of four main subject areas: Life Sciences (4200 journals), Health Sciences (6500 journals), Physical Sciences (7100 journals) and Social Sciences (7000 journals). These four areas of Science are further divided into 27 categories, which further include 300 subdisciplines. Moreover, journals may be classified under more than one discipline. The analysis in this paper was performed on the level of 26 Scopus major subject areas of science. These disciplines are the following: Agricultural and Biological Sciences (AGRI), Arts and Humanities (ARTS), Biochemistry, Genetics and Molecular Biology (BIOC), Business, Management and Accounting (BUSI), Chemical Engineering (CENG), Chemistry (CHEM), Computer Science (COMP), Decision Sciences (DECI), Dentistry (DENT), Earth and Planetary Sciences (EART), Economics Econometrics and Finance (ECON), Energy (ENER), Engineering (ENGI), Environmental Science (ENVI), Health Professions (HEAL), Immunology and Microbiology (IMMU), Materials Science (MATE), Mathematics (MATH), Medicine (MEDI), Multidisciplinary (MULT), Nursing (NURS), Pharmacology Toxicology and Pharmaceutics (PHAR), Physics and Astronomy (PHYS), Psychology (PSYC), Social Sciences (SOCI), Veterinary (VETE). Hereafter we use above initials to identify, except if stated otherwise, the considered disciplines. We also note that the Scopus database is well known for its lack of transparency in terms of the changes it performs to categories . The limits and scopes of Scopus, that also apply to this work, are explained in detail in its Content Coverage Guide (Elsevier 2017b).

**Fig. 1:**
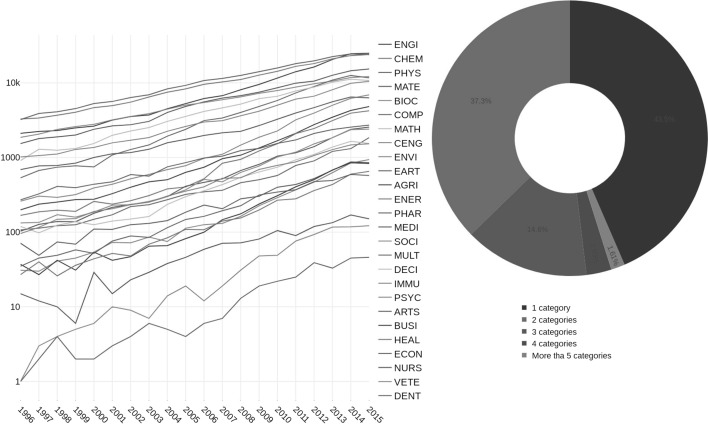
(Left) Number of publications for 26 different categories in the Scopus classification from 1996 to 2015 using the entropy search term. (Right) The pie chart depicts the number of papers belonging to 1, 2, 3, 4 and more than 5 categories in the Scopus classification. The full counting of papers was used. We observe that 43.5% of the papers belong to 1 category, 37.3% share 2 categories, 14.5% share 3 categories, and 2.5% reach 4 categories. See supporting information online at Figshare for a high resolution version of this figure

## Entropy with a given name

Several names have been associated to different definitions of entropy. In chronological order the first names are: Clausius (1865, Thermodynamics) (Clausius 1862), Boltzmann (1872, Kinetic Theory) (Boltzmann 1872), Gibbs (1878, Classical Statistical Mechanics) (Gibbs 1878), von Neumann (1927, Quantum Statistical Mechanics) (von Neumann 1927) and Shannon (1948, Theory of Communication) (Shannon 1948). In the Scopus database, the input “Clausius entropy” appears in the title or the abstract of 1917 documents, “Boltzmann entropy” in 15739 documents, “Gibbs entropy” in 31310 documents, “von Neumann entropy” in 8819 documents, and “Shannon entropy” in 30,194 documents. The Clausius and von Neumann entropies appear in a smaller number of documents because they are frequently referred as “thermodynamic entropy” (102456 documents) and “quantum entropy” (79253 documents), respectively. However, except for the Shannon entropy, if one looks for the citations of the seminal papers of these authors where the corresponding definition of entropy was introduced, previous numbers strongly diminish. We think that this fact is because either they are well-known, so they do not need to be cited, or they are indirectly cited by using other more general references, such as generic textbooks.

From considerations above and numerical values, it is clear that the entropy concept is very much alive in all branches of research. Figure 1 shows the continuous growth of publications along the analyzed years in all Scopus disciplines, not only in the expected categories as PHYS, CHEM, ENGI, BIOC, but also in seemingly distant fields as NURS, SOCI, and ARTS. As representative examples for the year 2015 it is seen that PHYS, ENGI and CHEM are above 23,000 citations while SOCI gets 2358 citations and ARTS 872 citations. Thus, it is clear that entropy and its applications play a central role in the career of modern scientists (Broadbridge and Guttmann 2009). According to the values shown on the vertical axis, a publication can be ascribed to one or more categories (see pie chart of Fig. 1) and we can see that around 55% of the journals are classified to more than one Scopus category, thus showing the great interdisciplinary nature of entropy research papers.

**Fig. 2:**
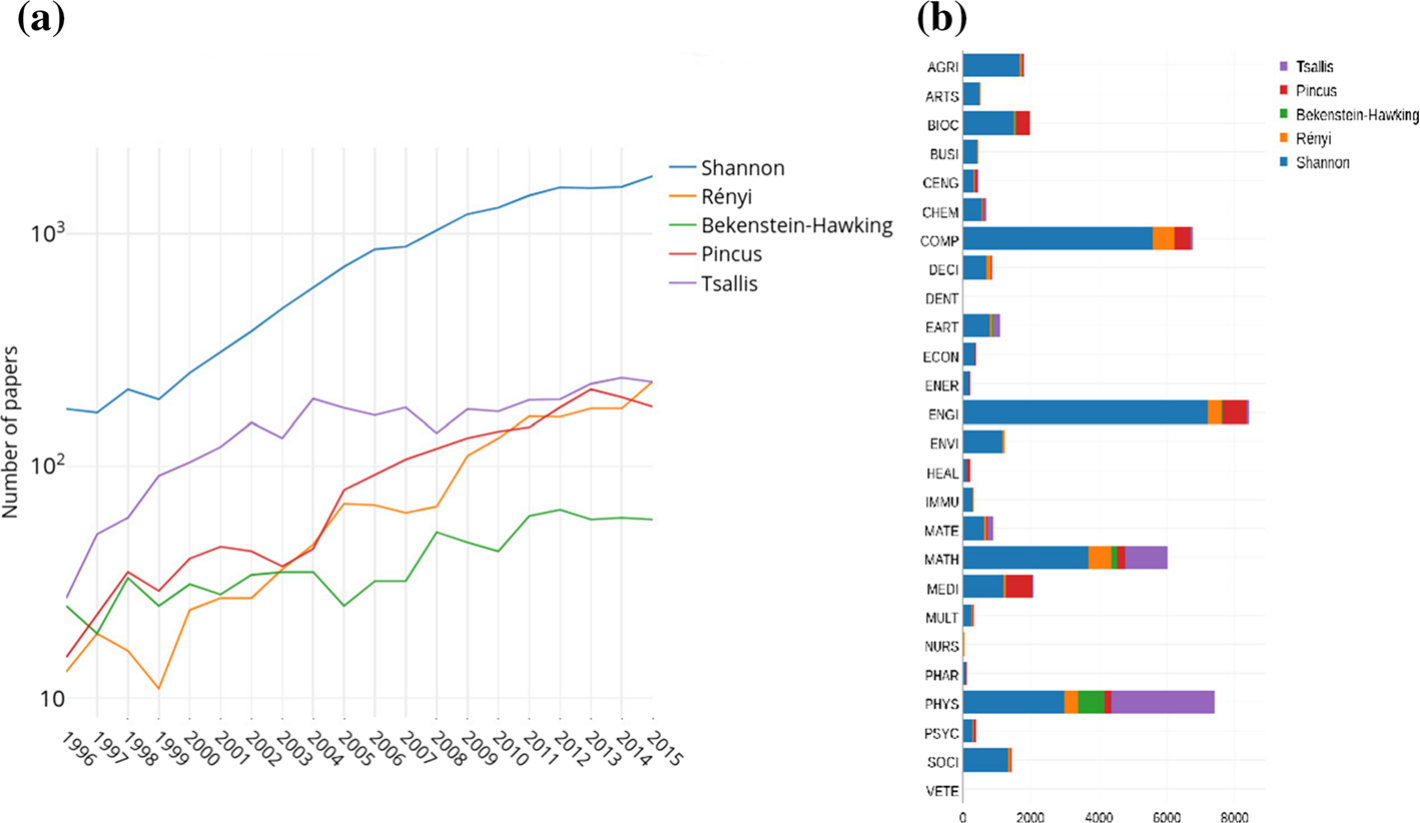
**a** Number of publications for 5 different entropies with given name in the Scopus database from 1996 to 2015. **b** Number of papers per category for 5 different entropies with given name. See supporting information online at Figshare for a high resolution version of this figure

In order to get a deeper insight, we now focus on some particular and significant meanings coming from those seminal papers which gave rise for first time to a named entropy and their impact on the different categories of the Scopus database. In this sense, the most cited papers in the period 1996–2015 are: Shannon (1948) (16600 citations), (Tsallis 1988) (3028 citations), (Pincus 1991) (1899 citations), (Rényi et al. 1961) (1641 citations) and (Bekenstein 1973) (800 citations). Figure 2a shows the yearly distribution of the citations of these papers. Note the high prevalence over the years of the use of Shannon’s entropy with a continuous increase of citations, while the other entropies with given name show, in general, a continuous but more moderate behavior.

The so-called Shannon entropy (first called “measure of information”) was proposed by Shannon (1948) in a paper concerning the average lack of information in a signal or message. The number of citations of Shannon paper increases from 176 citations in 1996 to 1777 citations in 2015. It is worth mentioning a book by Shannon and Weaver (1949) devoted to the mathematical theory of information, published in 1949, with 16145 citations. This book is frequently cited as a reference for the Shannon entropy instead of the seminal Shannon paper.

Tsallis (1988) proposed a “non-additive entropy”, known as Tsallis entropy, able to describe complex structures (such as fractals) using probabilities raised to a power. The number of citations of Tsallis paper increases from 27 citations in 1996 to a maximum of 240 citations in 2014, decreasing to 230 citations in 2015.

Pincus (1991) proposed “an approximate entropy”, known as Pincus entropy, to determine changing system complexity from a set of data distribution associated to both deterministic and chaotic processes, such as experimental time-series data. The number of citations of Pincus’s paper increases from 15 citations in 1996 to a maximum of 214 citations in 2013, decreasing slightly in the two last years: 198 citations in 2014 and 180 citations in 2015.

Rényi et al. (1961) proposed “the entropy of order *q*” of a distribution, known as Rényi entropy, in order to extend the Shannon entropy as a measure of information. The number of citations of Rényi paper increases from 13 citations in 1996 to a maximum of 231 citations in 2015, surpassing the number of citations of the entropies of Tsallis and Pincus.

In 1972, in order to formulate the second law of thermodynamics in a suitable form for black hole physics, Bekenstein (1973) provided an expression for the entropy of a black hole, nowadays usually known as Bekenstein–Hawking entropy (Hawking 1971). The number of citations of the Bekenstein paper increases, with small fluctuations, from 25 citations in 1996 to 59 citations in 2015. A related point to consider is the existence of a Bekenstein’s article devoted to the black hole entropy, published in 1973 (Bekenstein 1973) with 2946 citations. This paper is frequently cited as a reference for the black hole entropy instead of the seminal Bekenstein paper published in a less relevant journal.

Figure 2b shows the number of citations of these seminal papers per category. This figure clearly shows the significance of the use of each entropy in each category which is related to the corresponding definition. The number of citations in categories is always greater than the citations of the papers because, as aforementioned, one journal can be assigned to several categories (see inset in Fig. 1). For the considered papers, the order in the number of citations per category is also maintained: Shannon (31882 citations), Tsallis (4957 citations), Pincus (3401 citations), Rényi (2856 citations) and Bekenstein (1039 citations). However, the order by the number of categories in which these seminal papers are cited is as follows: Shannon and Pincus (26 categories), Rényi (25 categories), Tsallis (14 categories) and Bekenstein (12 categories). Ten categories (BIOC; CENG; CHEM; COMP; EART; ENGI; MATE; MATH; MEDI; and PHYS) include citations of the five seminal papers.

The Shannon paper is the most cited paper in 25 categories (except in PHYS) being ENGI the top category (7205 citations) followed by COMP (5562 citations). The Tsallis paper is the most cited paper in PHYS (3026 citations), followed by MATH (1260 citations). For the Pincus paper, MEDI is the top category (763 citations) followed by ENGI (694 citations). For the Rényi paper, MATH is the top category (670 citations) followed by COMP (653 citations). Finally, for the Bekenstein paper, PHYS is the top category (784 citations) followed by MATH (170 citations). We note in Fig. 2b the relatively small number of citations in CHEM while this discipline is the second greater in publications in Fig. 1. A possible explanation of this fact could be that the usage of the entropy concept in CHEM is mainly associated to the Clausius (i.e., thermodynamics) definition which, as stated above, is not explicitly cited but indirectly and/or using generic books references.

## Entropy-related key concepts

**Fig. 3:**
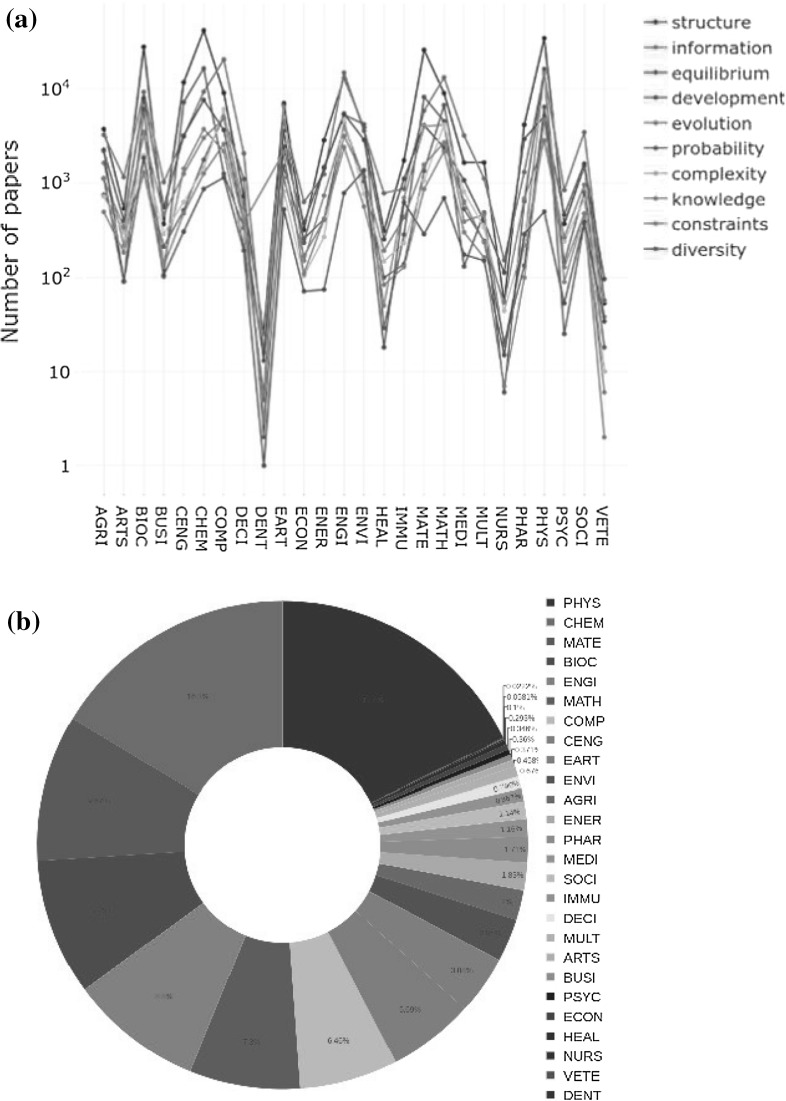
**a** Number of papers per category for ten key entropy concepts. The concepts were selected according to their frequency of appearances in all abstracts in our dataset. **b** Percentage of number of papers published in each category for the entropy key concepts. See supporting information online at Figshare for a high resolution version of this figure

Entropy’s original meaning in Thermodynamics, mainly linked to equilibrium, irreversibility and/or time evolution, was associated to energy loss (i.e., degradation) in fields related to heat devices. Later, Boltzmann (1872) and Gibbs (1878) extended entropy on a statistical basis and it was associated with probability and order/disorder in physical systems, and in living systems by Schrödinger (1944). After Shannon’s works (Shannon 1948; Shannon and Weaver 1949; Shannon 1951), the information entropy was firstly defined as a measure of the amount of information in a transmitted text, and it is considered as a measure to eliminate uncertainty in a message which needs information to become ordered. In the last decades there are many new related concepts of entropy on both theoretical and computational aspects. This is mainly derived from the massive use of computers which has allowed the unifying analysis of entropy in many complex systems of very different nature (social, environmental, geographic, agricultural, ocean, linguistic, urban, biomolecular, financial, forecasting, and so on) and with scales ranging from microscopic to cosmological (Rao et al. 2016; Phillips et al. 2006; Srivastav and Simonovic 2015; Osgood et al. 2016; Hirsh et al. 2012; Almeida 2001; Cabrera et al. 2017; Bekiros et al. 2017; Veríssimo et al. 2017).

As a logic consequence, the related entropy concepts have moved in order to fit this new research in complex systems and the different involved scales. We have looked in the abstracts and titles of the considered papers the number of appearances of these new concepts. We have found that the most cited entropy related key concepts, listed in descending order, are: *structure, information, equilibrium, development, evolution, probability, complexity, knowledge, constraints, diversity, dispersion, degradation, disorder, dissipation, irreversibility, and intelligibility*.

It is illustrative to link the number of appearances of the above entropy related key concepts to the different categories in the Scopus classification. The results of such a study are presented in Fig. 3a for the top ten concepts. Beyond the particular behavior of each concept in each category (which can be checked in the supplementary material), some general comments are in order:

(a) Clearly, *Structure* is the most important entropy related key concept, not only in the absolute number of appearances but also by its presence in all analyzed categories. Especially useful are their maximum values, as expected, in the categories CHEM, BIOC, PHYS, and MATE. We also stress its non negligible influence on categories, with a priori scarce physical content such as BUSI, ECON, and even in ARTS and SOCI.

(b) On a second and similar level appears *Information* and *Equilibrium*. The former shows a high number of appearances in COMP and MATH, while the maximum values in the latter coincide mostly with those seen above for the *structure* concept.

(c) On a lower level by number of appearances, but more evenly distributed among different categories, are the remaining seven concepts: *development, evolution, probability, complexity, knowledge, constraints, and diversity*. The lattermost deserves a comment: it appears in different meanings, mainly as a biodiversity index in biology and related areas (AGRI, ENVI, IMMU), though its cultural meaning is also observed in BUSI, and ECON disciplines.

The observed highly peaked structure of the above figure around some categories is highly consistent with the number of papers published in each involved category, as can be checked in Fig. 3b, where we show this magnitude (in percentage).

**Fig. 4:**
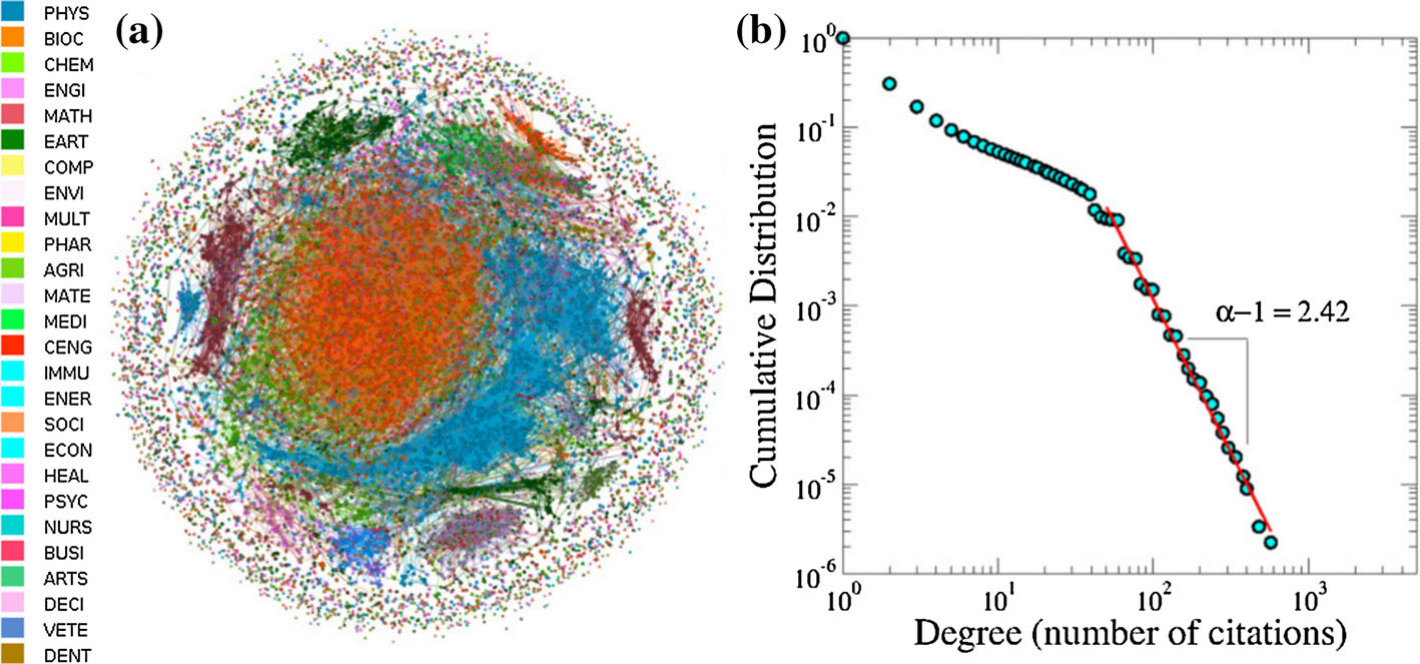
**a** Representation of the citation network associated with the *entropy* topic for publications during the period 1996–2015. Here, nodes represent publications and links refer to the citations. The colors in nodes correspond to the categories listed on the left. **b** Complementary cumulative distribution of the citation network in our study. The power-law exponent was estimated for the tail in the distribution by means of the MLE method (Clauset et al. 2009). The obtained value is $\alpha -1=2.42\pm 0.01$. See supporting information online at Figshare for a high resolution version of this figure

**Fig. 5:**
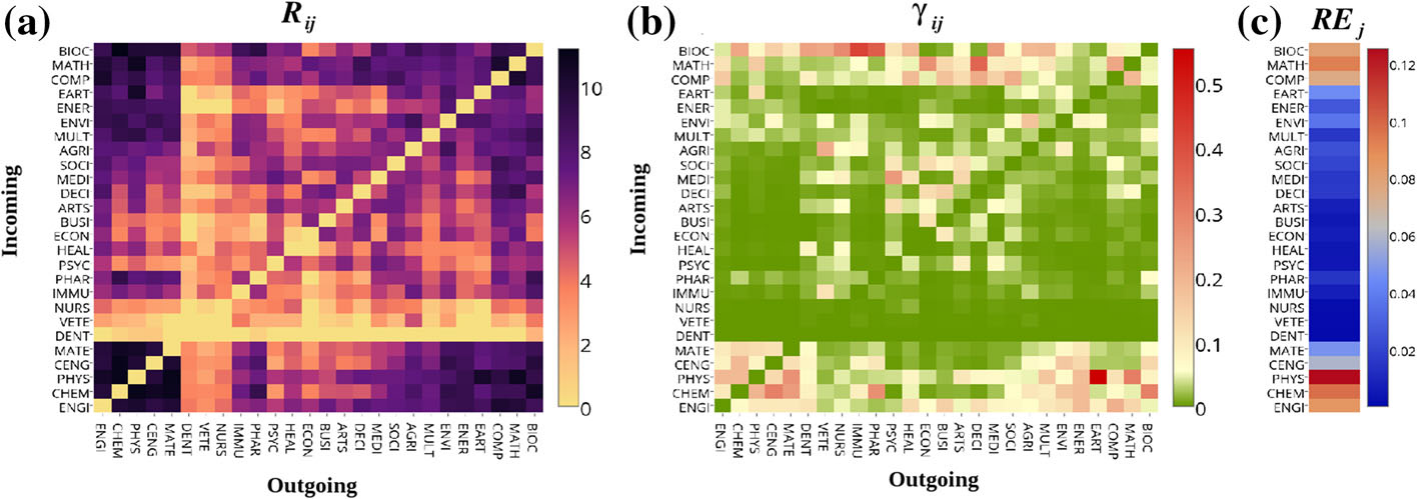
**a** Representation of the matrix ($R_{ij}$) of outgoing (horizontal axis) and incoming (vertical axis) citations between all pairs of categories. The color bar represents (in log scale) the volume of incoming or outgoing citations. **b** Matrix ($\gamma _{ij}$), which represents the normalized data showed in **a** by the total number of citations given by publications in category *i* to all other categories. **c** Values of $RE_j$, which represents the relative external importance of category *j*. See supporting information online at Figshare for a high resolution version of this figure

## Categories interaction: citations, papers classification and key concepts

In this part of our study, we investigate the cross-disciplinary knowledge transfer in terms of the emerged networks from the collected database. In particular, we are interested in evaluating the structure of the research category space based on interactions derived from (i) citations among papers from different categories (ii) distribution of papers according to the Scopus classification; and (iii) appearance of key entropy concepts in abstracts and titles.

**Fig. 6:**
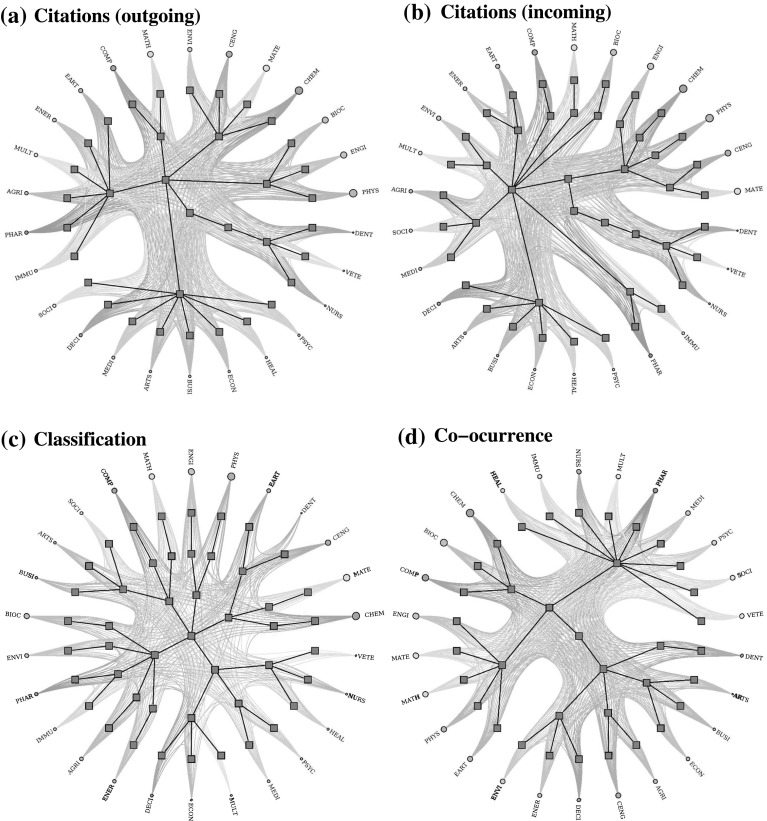
Representation of hierarchical structure of categories based on distribution of papers in categories. The size of the nodes correspond to the total number of publications. See supporting information online at Figshare for a high resolution version of this figure

(i) Regarding (i), and prior to our analysis of category interaction, we present a brief description of the citation network under study. A visual representation of the citation network is depicted in Fig. 4a, where nodes represent publications and links denote citations. For visualization purposes, we have colored the nodes according to the category to which each article belongs, and we have excluded publications that belong to 2 or more categories. As it is shown in Fig. 4b, the complementary cumulative distribution is well described, for large degree values (citations), by a power-law function of the form $G(>x)\sim x^{-(\alpha -1)}$, with $\alpha$ a scaling exponent related to the probability function $p(x)\sim x^{-\alpha }$. To calculate $\alpha$, we used the maximum likelihood estimation (MLE) method (Clauset et al. 2009), which yielded $\alpha -1=2.42\pm 0.01$. This value for $\alpha$ is in good concordance with the scaling exponent ($\alpha ^{{*}} \approx 3.54$), reported for the citation probability distribution of citations between articles from all subject areas in the *Scopus* database (Brzezinski 2015; Albarrán and Ruiz-Castillo 2011).
  A visual representation of the values of the matrix $R_{ij}$, which represents the network flow in terms of incoming and outgoing citations (see “Appendix” for details), is depicted in Fig. 5a. To indicate the importance of each row/column (category), the color panel (in $\log$ scale) represents the volume of citations incoming or outgoing. Some aspects worth noticing are the great volume of citations between the categories ENGI, CHEM, PHYS, CENG and MATE; the interaction PHYS-CHEM (in reciprocal directions) is the one that exhibits the strongest ties, while the other interactions between these categories show moderately high interaction with dominant participation of CHEM. We also notice that this group (ENGI, CHEM, PHYS, CENG and MATE) shares a large number of incoming citations with BIO, MATH, COMP, EART and MULT. Similar conclusions can be obtained for the interaction of COMP and MATH with a reciprocal level of citations by these categories. We observe a quite different situation for the relation between categories that belong to Life, Social and Health Sciences, as they exhibit similar intermediate mutual relationships; Physical Sciences represents its main source of information in terms of the entropy-related key concepts.
  When $R_{ij}$ is normalized by $R_i$, i.e., $\gamma _{ij}$ is considered, the behavior of the matrix reveals the categories with a tendency to receive information from a specific category (see Fig 5b). Here, we observe that EART and IMMU are the categories with a high tendency to cite articles from specific disciplines such as PHYS and BIOC, respectively. It is also worth noting that disciplines from Physical Sciences exhibit intermediate values with reciprocal behavior, indicating that the knowledge transfer occurs in both directions with a similar intensity, while the interaction of Physical Sciences with Life, Social and Health Sciences is clearly asymmetrical with dominant direction of the knowledge flow from the former to the latter.
  The results of $RE_j$ are shown in Fig. 5c. We observed that categories like PHYS, CHEM, ENG, MATH, BIOC and COMP, are by far the most relatively influential on many categories, however in a lower importance scale are CENG, MATE and EARTH. Besides, disciplines from Health and Social Sciences are the less influential categories.
  In order to further explore the interaction of categories in terms of citations, we notice that the configurations that emerge when considering the citation flow reveals a dense distribution of links between the categories, making necessary the use of methods of segmentation to identify the potential presence of modules (Rosvall and Bergstrom 2008). The accurate detection of modules permits to analyze the observed system in terms of their building blocks which exhibit a clear tendency to be internal-densely connected compared to the number of links with the rest of the network, providing key information about their organization. Here we adopt the methodology proposed in Peixoto (2014), which consists in detecting hierarchical structures by means of a variant of a stochastic block model (see “Appendix” and ref. Peixoto 2014 for details). This procedure has been used widely as a representative model to study the fragmentation of a network into clusters or groups of nodes that are more densely connected (Fortunato and Hric 2016).
  As stated above, the connectivities in terms of citations can be separated into outgoing and incoming links, yielding two representations of the network. As shown in Fig. 6a, the application of the nested stochastic block model reveals that, for the outgoing citation network, categories are separated into two sets at the topmost inner level, while for intermediate divisions the segmentation block model detects 6 groups with a dense distribution of connections between blocks. Here, the size of each node or category (circles in the figure) represents the total number of publications in the category. We notice that DENT, VETE and NURS are categories which remain as independent from almost the most basic level, indicating that papers in these categories usually cite publications among them, but there is not predominance in the citations the rest of categories. We also point out that, at the next level, the other branch subdivides into 5 groups which exhibit a clear segregation related to the dominance of correlations between citations among the categories. These remaining 5 groups identified at the intermediate level have various numbers of categories ranging from 2 to 8, indicating a different structure of connectivities. For instance, MATH and COMP (top-left side in Fig. 6a) are members of a single community whereas PSYC, HEAL, ECON, BUSI, MEDI, ARTS, DECI and SOCI (lower side in Fig. 6a) represent the largest one.Clockwise, the other 3 clusters are formed, respectively, by ENVI, CENG, MATE, and CHEM; BIOC, ENGI and PHYS; IMMU, PHAR, AGRI, MULT, ENER, and EART.
  For the incoming citation network (Fig. 6b), the segmentation procedure revealed a different picture since the fragmentation at the inner topmost level revealed a separation between 2 groups: a small one (DENT, VETE and NURS) and the rest of the network. At the next level, the largest group subdivides into 2 blocks, where ENGI, CHEM, PHYS, CENG and MATE (top-right side) form a single group with a high tendency to be cited themselves and, on a minor scale cited by other categories as well; the second subgroup is formed by categories belonging mostly to Social Sciences. At the intermediate level, this second group is subdivided into 8 modules. We notice that MATH, COMP and BIOC are classified as independent categories from the intermediate level, whereas the other subgroups are comprised of 2 to 6 categories. These modules are formed by (counter-clockwise in Fig. 6b) EART and ENER; ENVI and MULT; AGRI, SOCI and MEDI; IMMU and PHAR; and DECI, ARTS, BUSI, ECON, HEAL and PSYC.
(ii) For the point (ii) regarding the distribution of papers in categories, as it was shown in Fig. 1, nearly 60% of the papers belong to 2 or more categories, indicating that the majority of published works can be identified or related to different areas of Science. Since research articles belong to different categories at the same time, an interaction between categories can be defined in terms of the number of publications they share. This relationship is representative of proximity between areas according to the Scopus classification. We also apply the nested block model (Peixoto 2014) to the network of categories and the results are depicted in Fig. 6c. At the inner topmost level the block model identifies five groups which contain the following categories (listed in clockwise): ENGI and PHYS for group 1; EART, DENT, CENG, MATE, and CHEM for group 2; VETE, NURS, HEAL, PSYC, MEDI, SOCI,MULT, ECON, and DECI for group 3; ENER, AGRI, INMU, PHAR, ENVI, and BIOC for group 4; BUSI, ARTS, SOCI, COMP, and MATH, for group 5. When looking at the internal structure of these four groups, it is observed that new partitions are determined, leading to 16 subgroups at the next level, and culminating in 26 categories. We notice that for the intermediate level, there are some categories which tend to share more articles with other groups whereas other categories mostly connect with specific categories. Moreover, it is observed that group 5 clearly separates from the others at the very topmost level, indicating that articles in these categories tend to be classified as single category papers.
(iii) Concerning the appearance of entropy-related key concepts in abstracts and titles, point (iii), we resort to the use of natural language processing techniques to identify these key concepts in texts (see “Appendix”). The analysis of the interaction content in terms of concepts or phrases may provide valuable information on knowledge transfer from one discipline to another. In this case, the interaction between categories is dictated by the occurrence of key concepts in titles and abstracts. Specifically, a connection between two articles from different disciplines exists if the two papers share a common key concept. In this way, a link between the two categories to which the papers belong is defined. Figure 6d shows the results of the partition procedure for the shared key concept network. We observe that a different subdivision is present at the topmost level, with 2 basic groups which contain subgroups that remarkably relate categories of the Physical Sciences with categories of the Biological Sciences and Engineering (e.g., CHEM and BIOC; PHYS and ENGI). In a second intermediate level 7 subgroups are observed. For this structure, it is worth mentioning that some categories, which are frequently assumed to be close in terms of citations, are not in the same subgroup, i. e., do not necessarily share an important number of the key concepts. For instance, COMP shares key concepts with BIOC and CHEM, whereas in terms of citations COMP has important ties with MATH.

## Discussion and conclusions

The temporal evolution of papers from 1996 to 2015 clearly demonstrates a stronger increase of citations in all categories, especially those related to Economics, Engineering, and Biochemistry, although the importance of entropy in the Social Sciences is also very significant. In general, these results highlight the great impact of the concept of entropy in all branches of scientific inquiry. The results also demonstrate that concepts based on Shannon’s entropy have been the main driver in the rapid and expansive diffusion of entropy (first restricted to Thermodynamics) in all scientific fields through the widespread use of computers. This led to new concepts (such as structure, information, complexity, diversity, etc.) now used according to different scales and thematics from Physics and Chemistry to disciplines of Social Sciences and Humanities, including Economics and Information Science.

Moreover, the dissemination of entropy-based concepts has caused the emergence of entropies with a given name, i.e., increasing the eponyms that identify particular new definitions. The results obtained show that categories in Natural Sciences are most relevant in terms of the number of citations, i.e., as a source of knowledge; in particular, PHYS, CHEM, and ENGI (as expected) were not the only categories to play an important role in the dissemination of the concept of entropy in all areas of research, but also MATH, BIOC, and COMP are quite relevant in this dissemination process. Conversely, the disciplines of Health and Social Sciences are the least influential categories.

Here, we have explored the interaction between disciplines from the different perspectives that reveal different patterns of interconnection. With citations, classification of articles and co-occurrence information, it is possible to compare the interaction between disciplines with the identification of strong and weak links. The proximity between disciplines can be defined by the use of the same citations and the co-occurrence of key words in the abstracts and titles. The Scopus categories themselves do not contain relevant information on the proximity between its categories. Previous work has highlighted the importance of maps of science based on journal articles with descriptions of local and global structure (Boyack et al. 2005). In our case, the interaction between areas of science were analyzed with information restricted to documents containing the word “entropy“, thus, resulting maps only reveal the interconnections of this subset, which is a relatively small number of articles compared to the total number of scientific publications. The results of the application of the stochastic block model to disciplines based on citation patterns (incoming and outgoing) are in general concordance with a recent study of a hierarchical block structure with different levels of resolution, from journals to fields of knowledge (Hric et al. 2017). The hierarchical structures that emerge in our segmentation reveal that more important subgroups share disciplines such as PHYS, CHEM, MATE, CENG, BIO, MATH y COMP, which means that these areas of science are sources and targets of citations.

In summary, we have presented a bibliometric study of the interdisciplinary characteristics of entropy and the related cross-disciplinary knowledge transfer by using categories provided by the Scopus database. It is worth noting that the results of the segmentation for the three forms of interaction (citations, classification, and co-occurrence of concepts) show that different levels of proximity exist between categories, which concords with recent studies based on a linguistic similarity approach (Dias et al. 2017). Finally, we would like to remark that potential sources of bias in our study are the lack of completeness of the retrieved database and the lack of transparency in the categorization performed by Scopus.

## Appendix: Methods

*Citations between categories*      In order to evaluate quantitatively the interdisciplinary impact of the entropy concept, the following quantities are useful for our description (Egghe and Rousseau 2000; Rinia et al. 2002):

- The share number of citations given by articles in category *i* to publications in category *j*: $R_{ij}$.
- The share number of citations given by articles in category *i* to publications in category *j* (normalized by the total number of citations given by publications in category *i*)
  $$\gamma _{ij}=\frac{R_{ij}}{R_i},$$
  where $R_{i}=\sum _j R_{ij}$ represents the total number of citations given by publications in category *i* to all other categories.
- The relative external importance of category *j* is given by Rinia et al. (2002),
  $$RE_j=\sum _{i (\ne j)} \gamma _{ij}(\beta _i)(1/\beta _j),$$
  where $\beta _i$ and $\beta _j$ represent the size (number of publications) of citing and cited category, respectively.

We notice that the definitions of $\gamma _{ij}$ and $RE_j$ are constructed as indicators of the relative importance of category *j* with respect to all other disciplines and reflect the openness of all other categories to the specific category *j* (Rinia et al. 2002); we recall this point because we are only interested in exploring the interaction of categories, which is why self-citations are excluded.

*Hierarchical stochastic block model* The hierarchical block model used for the segmentation of the networks is based on the edge counts among nodes (Peixoto 2014). As a generative model, it works with the assumption that the network has as many blocks as nodes in the first level, changing as the edge count creates blocks of nodes with stronger ties. The next level of the hierarchy is always based on the one before and thus, the hierarchy is generated recursively until only one block is left. Moreover, the degree corrected version is used, which means that the degree sequence of the node’s network is used as an extra parameter in the block generation, which has been proved to yield better results on empirical data (Peixoto 2012).

The model is based on the classic stochastic block model (Holland et al. 1983; Faust and Wasserman 1992; Anderson et al. 1992), which considers *N* nodes, divided into *B* blocks, with $e_{ij}$ edges between blocks *i* and *j*, respectively. Then, the edge counts, $e_{ij}$, are the parameters for the model. So, we are using the connection between nodes to generate the blocks. Because the nested version is used (Peixoto 2014), we have a multigraph for each block, where the nodes are the blocks and the edge count the edges of the new multigraph. This new multigraph is created with the same model as the previous one, hence, we have a recursive block model that will end up with a one block multigraph.

Then, for the first level of the model, $l=0$, which is the one working with the graph itself, where $E=\frac{1}{2}\sum _{ij} e_{ij}$ is the total number of edges, $N_k$ is the total number of nodes with *k* degree; finally, we have that the value of the entropy for such an ensemble,

$$\mathcal{S}_c \simeq -E - \sum _k N_{k}\ln {k!} - \frac{1}{2}\sum _{ij} e_{ij}\ln {\left( \frac{e_{ij}}{e_{i}e_{j}}\right) },$$

where $e_{i}=\sum _j e_{ij}$ stands for the number of edges that go to from block *j* to blocks *i*. This inference model creates *B* blocks, that are going be the nodes in the next level $l+1$, which are connected between themselves through the edge counts $e_{ij}$. After we calculate the entropy for the given model, we have to choose from the different possible configurations, the one that optimizes the entropy $\mathcal{S}_c$, which will be the final block structure of our hierarchy.

*Natural language processing* The titles and abstracts were analyzed in order to obtain information concerning the word entropy. The Natural Language Toolkit (Steven Bird and Loper 2009) was used to extract the noun phrases from each title and abstract. The noun phrases are sets of words that are related to a specific topic, or main idea, which matches the purpose of identifying the entropy concept inside the text. Then, we put together all abstract and title information for each category. The most counted words from the noun phases were used in order to extract a list of words that appeared the most in each category. The top ten of those words were finally selected, as appears on Fig. 3.
